# Supplementary figures and images for: Field testing an “acoustic lighthouse”: Combined acoustic and visual cues provide a multimodal solution that reduces avian collision risk with tall human-made structures
Source: PLoS One. 2021 Apr 28;16(4):e0249826. doi: 10.1371/journal.pone.0249826 (PMC8081207; doi:10.1371/journal.pone.0249826)

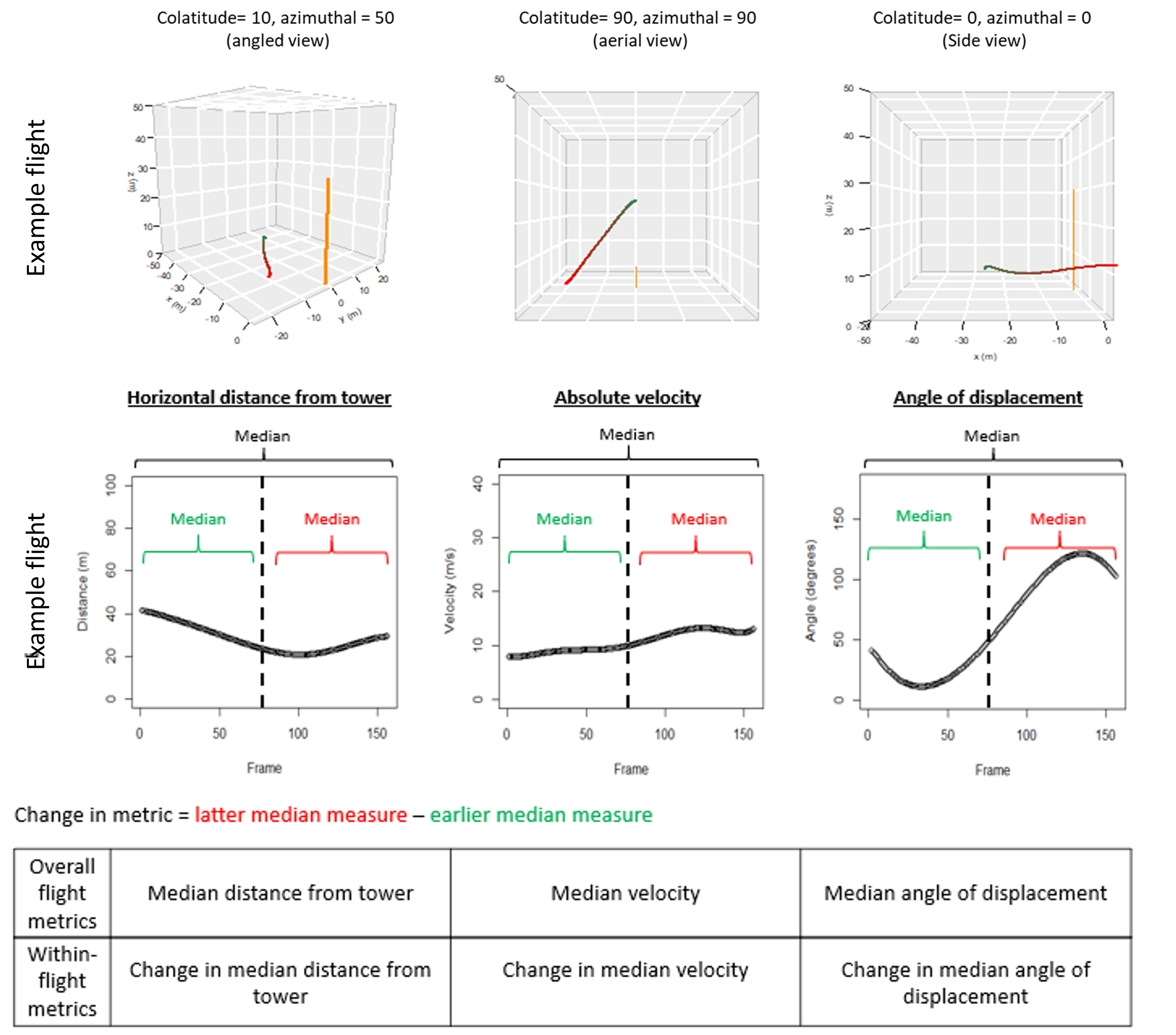

Supplement: S1 Fig — The top row of graphs illustrates a smoothed 3D reconstruction of a bird flight path around a tower. Flight behavior was characterized using measures of horizontal distance from the tower (d), absolute velocity (v), and horizontal displacement angle from the tower (ϴtower). These measures were summarized for an entire flight path using the median. Changes in flight behavior over the course of a bird’s flight were summarized using the change in the median from the earlier to latter half of the bird’s flight. (TIF) [file pone.0249826.s001.tif]

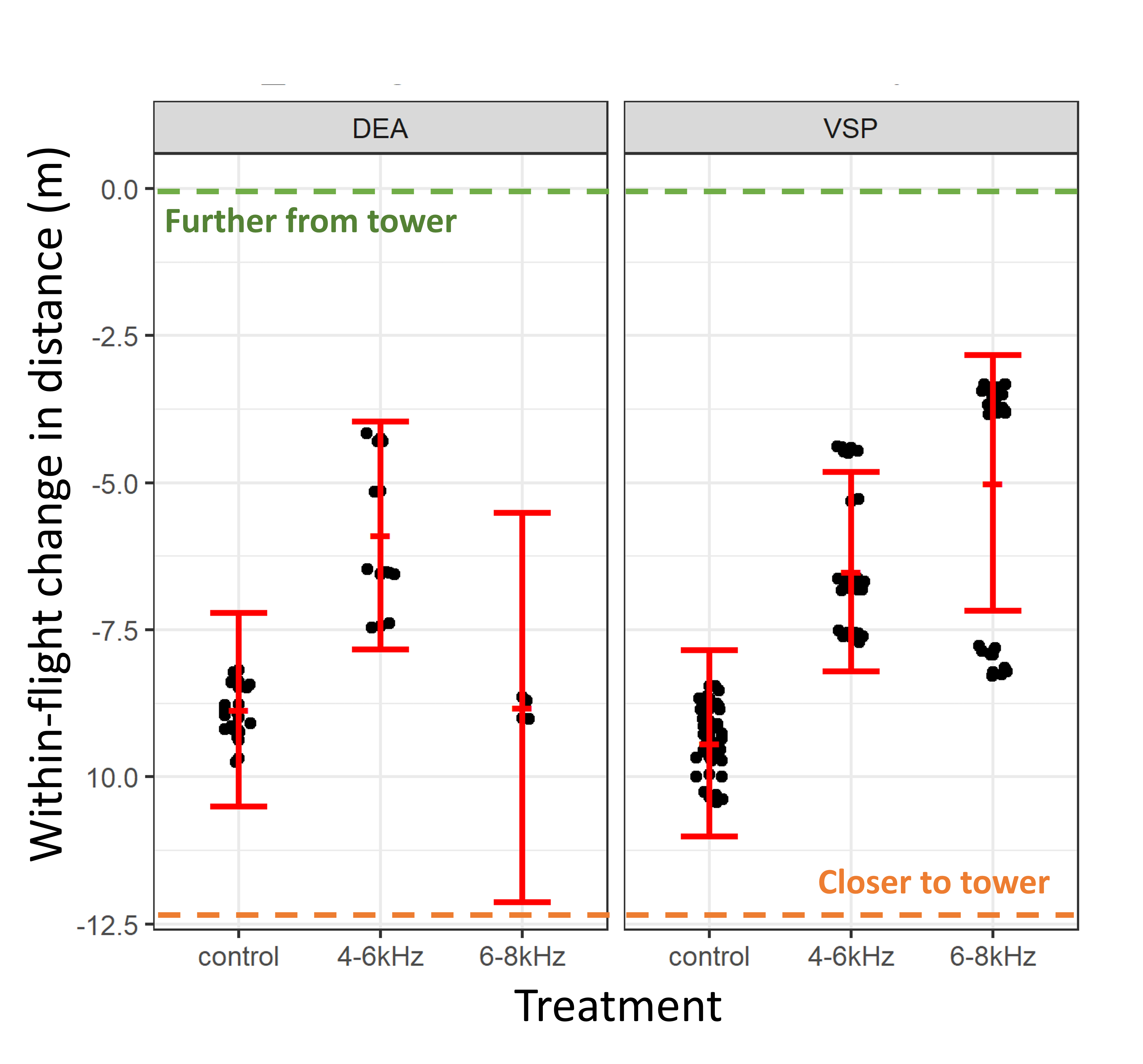

Supplement: S2 Fig — Change in distance by treatment condition within each tower site. Solid red dots and error bars represent model-averaged mean of outcome variable ± standard error of the mean. Green dotted line indicates a level of change in distance where flights remain further away from the tower. Orange dotted line indicates a level of change in distance where flights draw closer to the tower. (TIF) [file pone.0249826.s002.tif]

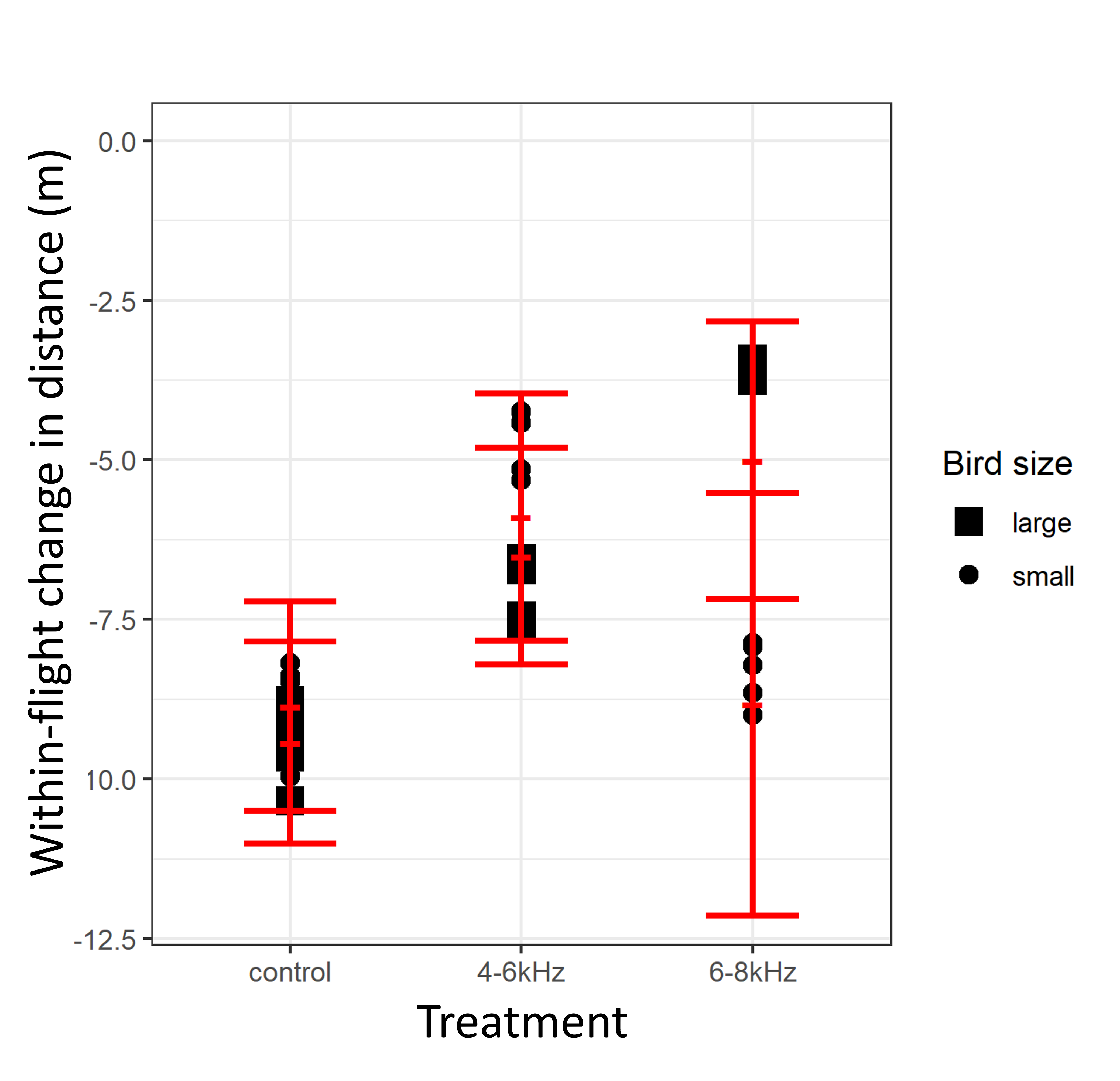

Supplement: S3 Fig — Solid red dots and error bars represent model-averaged mean of outcome variable ± standard error of the mean. Symbols indicate the attribute of bird size for each data point. (TIF) [file pone.0249826.s003.tif]

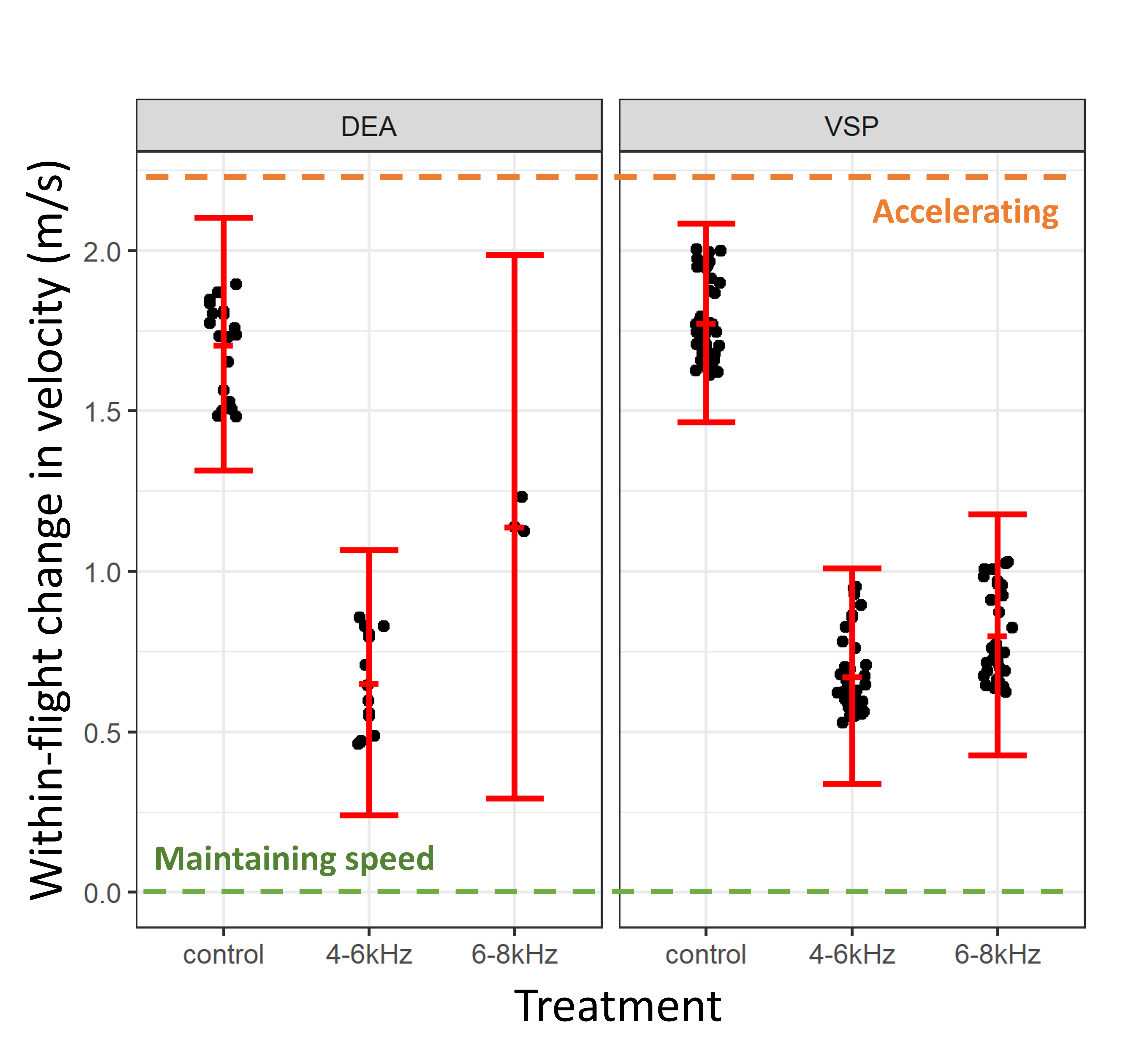

Supplement: S4 Fig — Change in velocity by treatment condition within each tower site. Solid red dots and error bars represent model-averaged mean of outcome variable ± standard error of the mean. Green dotted line indicates the level of change in velocity representing a maintaining of flight speed. Orange dotted line indicates the level of change in velocity representing an acceleration in flight speed. (TIF) [file pone.0249826.s004.tif]

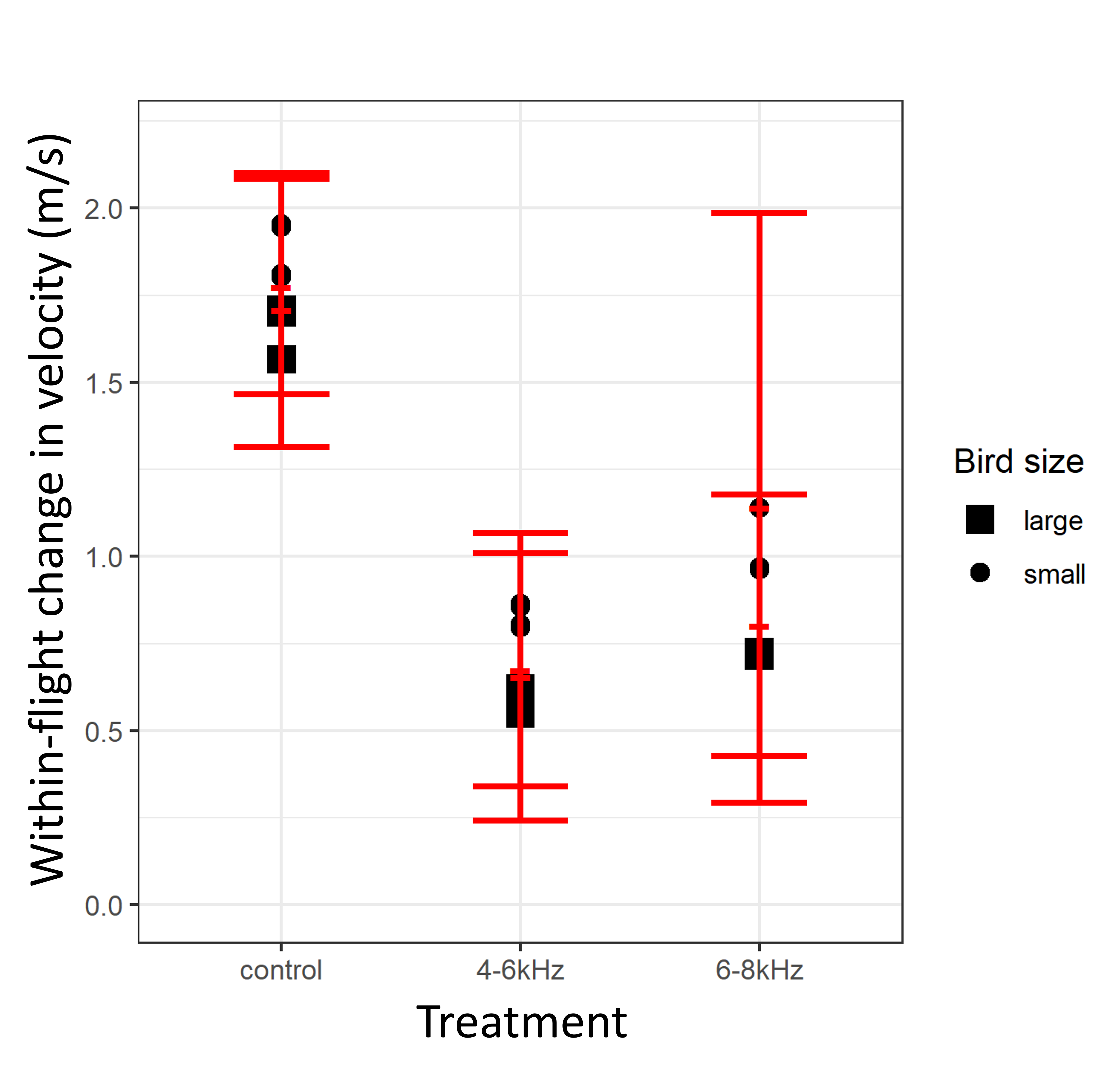

Supplement: S5 Fig — Solid red dots and error bars represent model-averaged mean of outcome variable ± standard error of the mean. Symbols indicate the attribute of bird size for each data point. (TIF) [file pone.0249826.s005.tif]

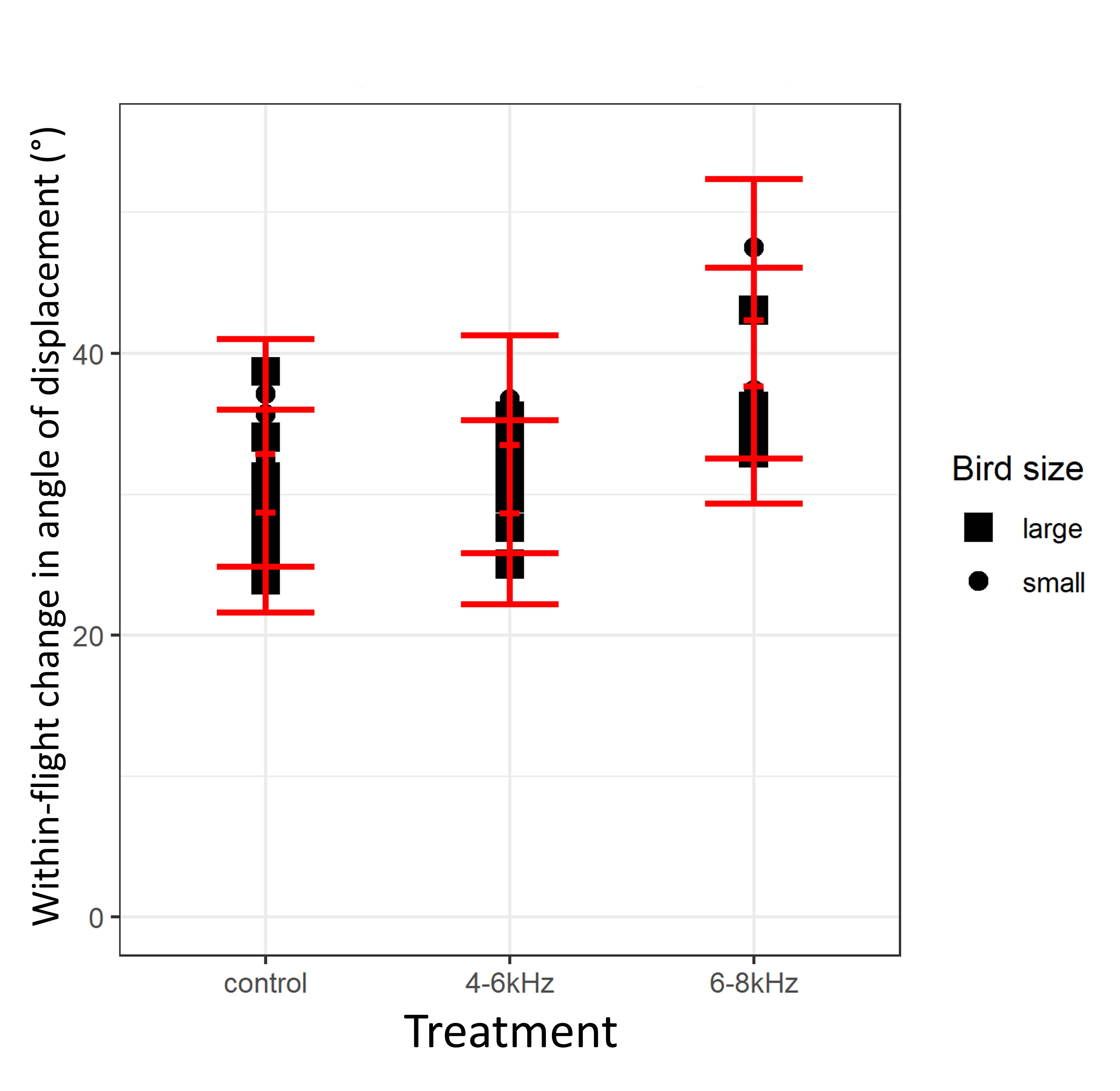

Supplement: S6 Fig — Solid red dots and error bars represent model-averaged mean of outcome variable ± standard error of the mean. Symbols indicate the attribute of bird size for each data point. (TIF) [file pone.0249826.s006.tif]
