# Supplementary material for: Field testing an “acoustic lighthouse”: Combined acoustic and visual cues provide a multimodal solution that reduces avian collision risk with tall human-made structures
Source: PLoS One. 2021 Apr 28;16(4):e0249826. doi: 10.1371/journal.pone.0249826 (PMC8081207; doi:10.1371/journal.pone.0249826)
Supplement: S1 Table — The same set of candidate models was applied to all outcome flight behavior metrics. Structure of linear predictors was based on a-priori hypotheses and exploration of non-linearity between predictors and response variables and of collinearity between predictor variables. (DOCX) [file pone.0249826.s007.docx]

**S1 Table. Set of candidate models.**

| Candidate model linear predictor |
| --- |
| 1 |
| treatment |
| site |
| date |
| treatment + site |
| treatment + date |
| site + date |
| treatment + site + treatment*site |
| treatment + date + treatment*date |
| site + date + site*date |
| treatment + site + date |
| treatment + site + date + site*date |
| treatment + site + date + treatment*site |
| treatment + site + date + treatment*date |
| treatment + site + date + treatment*site + treatment*date |
| treatment + site + date + treatment*site + treatment*date + site*date |
| treatment + bird_size |
| treatment + bird_group |
| site + bird_size |
| site + bird_group |
| treatment + site + bird_size |
| treatment + site + bird_group |
| treatment + site + date + bird_size |
| treatment + site + date + bird_group |
| treatment + bird_size + treatment*bird_size |
| treatment + bird_group +treatment*bird_group |

The same set of candidate models was applied to all outcome flight behavior metrics. Structure of linear predictors was based on a-priori hypotheses and exploration of non-linearity between predictors and response variables and of collinearity between predictor variables.
