# Supplementary material for: Field testing an “acoustic lighthouse”: Combined acoustic and visual cues provide a multimodal solution that reduces avian collision risk with tall human-made structures
Source: PLoS One. 2021 Apr 28;16(4):e0249826. doi: 10.1371/journal.pone.0249826 (PMC8081207; doi:10.1371/journal.pone.0249826)
Supplement: S2 Table — AICc weight was used to rank model suitability. Models carrying 95% of total AICc weights were preserved and worse performing but more complex nested models were removed. (DOCX) [file pone.0249826.s008.docx]

**S2 Table. Overall distance final model set.**

| Model | ΔAICc | weight |
| --- | --- | --- |
| treatment + site + date | 0 | 0.245 |
| treatment + date | 0.246 | 0.217 |
| site + date | 1.019 | 0.147 |
| treatment + site + date + bird_group | 1.362 | 0.124 |
| date | 1.507 | 0.115 |
| treatment + site + date + bird_size | 2.138 | 0.084 |

AICc weight was used to rank model suitability. Models carrying 95% of total AICc weights were preserved and worse performing but more complex nested models were removed.
