# Supplementary material for: Field testing an “acoustic lighthouse”: Combined acoustic and visual cues provide a multimodal solution that reduces avian collision risk with tall human-made structures
Source: PLoS One. 2021 Apr 28;16(4):e0249826. doi: 10.1371/journal.pone.0249826 (PMC8081207; doi:10.1371/journal.pone.0249826)
Supplement: S3 Table — AICc weight was used to rank model suitability. Models carrying 95% of total AICc weights were preserved and worse performing but more complex nested models were removed. (DOCX) [file pone.0249826.s009.docx]

**S3 Table. Change in distance final model set.**

| Model | ΔAICc | weight |
| --- | --- | --- |
| treatment + bird_size + treatment * bird_size | 0 | 0.587 |
| treatment + date + treatment * date | 4.539 | 0.061 |
| treatment + date | 4.638 | 0.058 |
| treatment + site + treatment * site | 5.113 | 0.046 |
| treatment | 5.247 | 0.043 |
| treatment + site + date + treatment * site | 5.491 | 0.038 |
| treatment + site | 6.601 | 0.022 |
| treatment + site + date | 6.729 | 0.02 |
| treatment + bird_group | 7.279 | 0.015 |
| treatment + bird_size | 7.31 | 0.015 |
| treatment + site + date + bird_size | 7.844 | 0.012 |

AICc weight was used to rank model suitability. Models carrying 95% of total AICc weights were preserved and worse performing but more complex nested models were removed.
