# Supplementary material for: Field testing an “acoustic lighthouse”: Combined acoustic and visual cues provide a multimodal solution that reduces avian collision risk with tall human-made structures
Source: PLoS One. 2021 Apr 28;16(4):e0249826. doi: 10.1371/journal.pone.0249826 (PMC8081207; doi:10.1371/journal.pone.0249826)
Supplement: S4 Table — AICc weight was used to rank model suitability. Models carrying 95% of total AICc weights were preserved and worse performing but more complex nested models were removed. (DOCX) [file pone.0249826.s010.docx]

**S4 Table. Overall velocity final model set.**

| Model | ΔAICc | weight |
| --- | --- | --- |
| treatment + site + date + site * date | 0 | 0.842 |
| treatment + site + date + bird_size | 6.072 | 0.04 |
| treatment + site + date + bird_group | 6.787 | 0.028 |
| treatment + site + date | 7.392 | 0.021 |

AICc weight was used to rank model suitability. Models carrying 95% of total AICc weights were preserved and worse performing but more complex nested models were removed.
