# Supplementary material for: Field testing an “acoustic lighthouse”: Combined acoustic and visual cues provide a multimodal solution that reduces avian collision risk with tall human-made structures
Source: PLoS One. 2021 Apr 28;16(4):e0249826. doi: 10.1371/journal.pone.0249826 (PMC8081207; doi:10.1371/journal.pone.0249826)
Supplement: S5 Table — AICc weight was used to rank model suitability. Models carrying 95% of total AICc weights were preserved and worse performing but more complex nested models were removed. (DOCX) [file pone.0249826.s011.docx]

**S5 Table. Change in velocity final model set.**

| Model | ΔAICc | weight |
| --- | --- | --- |
| treatment + bird_size | 0 | 0.247 |
| treatment | 0.284 | 0.214 |
| treatment + site + bird_size | 1.425 | 0.121 |
| treatment + site + treatment * site | 2.061 | 0.088 |
| treatment + site | 2.252 | 0.08 |
| treatment + bird_group | 2.396 | 0.074 |

AICc weight was used to rank model suitability. Models carrying 95% of total AICc weights were preserved and worse performing but more complex nested models were removed.
