# Supplementary material for: Field testing an “acoustic lighthouse”: Combined acoustic and visual cues provide a multimodal solution that reduces avian collision risk with tall human-made structures
Source: PLoS One. 2021 Apr 28;16(4):e0249826. doi: 10.1371/journal.pone.0249826 (PMC8081207; doi:10.1371/journal.pone.0249826)
Supplement: S6 Table — AICc weight was used to rank model suitability. Models carrying 95% of total AICc weights were preserved and worse performing but more complex nested models were removed. (DOCX) [file pone.0249826.s012.docx]

**S6 Table. Overall angle of displacement final model set.**

| Model | ΔAICc | weight |
| --- | --- | --- |
| treatment + bird_size + treatment * bird_size | 0 | 0.709 |
| treatment | 3.88 | 0.102 |
| treatment + bird_group | 5.457 | 0.046 |
| treatment + bird_size | 5.996 | 0.035 |
| treatment + site | 6.023 | 0.035 |
| treatment + site + bird_group | 7.641 | 0.016 |

AICc weight was used to rank model suitability. Models carrying 95% of total AICc weights were preserved and worse performing but more complex nested models were removed.
