# Supplementary material for: Field testing an “acoustic lighthouse”: Combined acoustic and visual cues provide a multimodal solution that reduces avian collision risk with tall human-made structures
Source: PLoS One. 2021 Apr 28;16(4):e0249826. doi: 10.1371/journal.pone.0249826 (PMC8081207; doi:10.1371/journal.pone.0249826)
Supplement: S7 Table — AICc weight was used to rank model suitability. Models carrying 95% of total AICc weights were preserved and worse performing but more complex nested models were removed. (DOCX) [file pone.0249826.s013.docx]

**S7 Table. Change in angle of displacement final model set.**

| Model | ΔAICc | weight |
| --- | --- | --- |
| treatment + site + date + bird_group | 0 | 0.134 |
| date | 0.387 | 0.111 |
| treatment | 0.615 | 0.099 |
| treatment + site | 0.735 | 0.093 |
| site | 1.179 | 0.075 |
| site + date | 1.208 | 0.074 |
| treatment + site + date | 1.525 | 0.063 |
| treatment + date | 1.564 | 0.062 |
| treatment + bird_group | 1.588 | 0.061 |
| treatment + site + bird_group | 1.799 | 0.055 |
| treatment + site + bird_size | 2.481 | 0.039 |
| treatment + bird_size | 2.702 | 0.035 |

AICc weight was used to rank model suitability. Models carrying 95% of total AICc weights were preserved and worse performing but more complex nested models were removed.
