# Supplementary material for: Field testing an “acoustic lighthouse”: Combined acoustic and visual cues provide a multimodal solution that reduces avian collision risk with tall human-made structures
Source: PLoS One. 2021 Apr 28;16(4):e0249826. doi: 10.1371/journal.pone.0249826 (PMC8081207; doi:10.1371/journal.pone.0249826)
Supplement: S8 Table — Precipitation (mm), mean temperature (°C) and cloud cover (oktas) are reported. Average daily estimates of weather variables were extracted from the PRISM Climate Group gridded dataset (Oregon State University). (DOCX) [file pone.0249826.s014.docx]

**S8 Table. Weather variables across sampling dates.**

| Date | ppt (mm) | tmean (degrees C) | cloudcover (oktas) |
| --- | --- | --- | --- |
| 9/24/2019 | 0 | 25.9 | 2 |
| 9/25/2019 | 0 | 21.1 | 1 |
| 10/1/2019 | 0 | 21.8 | 3 |
| 10/2/2019 | 0 | 23.1 | 5 |
| 10/10/2019 | 0.99 | 16.8 | 4 |
| 10/11/2019 | 0 | 17.6 | 7 |
| 10/19/2019 | 0 | 11.5 | 8 |
| 10/20/2019 | 5.95 | 14.8 | 6 |
| 10/29/2019 | 0 | 17.4 | 7 |
| 10/30/2019 | 0 | 16.9 | 2 |
| 11/4/2019 | 0 | 8.7 | 1 |
| 11/5/2019 | 2.67 | 12.6 | 0 |

Precipitation (mm), mean temperature (°C) and cloud cover (oktas) are reported. Average daily estimates of weather variables were extracted from the PRISM Climate Group gridded dataset (Oregon State University).
